# Clinical, pathological, and molecular investigation of *Mycoplasma pulmonis*-induced murine respiratory mycoplasmosis in a rat (*Rattus norvegicus*) colony

**DOI:** 10.14202/vetworld.2017.1378-1382

**Published:** 2017-11-25

**Authors:** Saurabh Chawla, Sarita Jena, Balaji Venkatsan, Kuna Mahara, Nilanjan Sahu

**Affiliations:** 1 Department of Animal House, School of Biological Sciences, National Institute of Science Education and Research, Bhubaneswar, Odisha, India; 2 Department of Animal House, Institute of Life Sciences, Bhubaneswar, Odisha, India

**Keywords:** murine respiratory mycoplasmosis, *Mycoplasma pulmonis*, polymerase chain reaction, rat colony

## Abstract

**Aim:**

*Mycoplasma pulmonis* (MP) remains potentially important rodent pathogen causing murine respiratory mycoplasmosis (MRM) which may go undiagnosed due to its asymptomatic nature. In the present study, we carried out clinical, pathological, and molecular investigations of MP-induced MRM in a rat colony.

**Materials and Methods:**

Two female Wistar rats were observed to be diseased in animal facility of NISER, Bhubaneswar, and were kept in isolation for further investigation. Both the animals were found to be positive for MP after serological and molecular tests. Thereafter, whole rat colony comprising of 36 animals was segregated based on clinical symptoms and further sampled for histopathological, serological, and molecular investigations. Tracheal washing and infected lung tissue were collected during necropsy examination for DNA extraction. Molecular diagnosis was done by polymerase chain reaction (PCR) assay using species-specific primers.

**Result::**

Classical symptoms of MP-associated respiratory tract infection were observed in only 2 of 36 infected animals, and most of the animals were found asymptomatic to the disease; however, all the animals were found to be carrier after necropsy and PCR assay. Gross and histopathological finding suggested severe congestion of the lungs along with suppurative and necrotizing pneumonia. The disease is confirmed by molecular diagnosis using species-specific primers in PCR assay.

**Conclusion:**

MRM may go undiagnosed due to asymptomatic nature. Detailed study of clinical symptoms, pathology, serology, and PCR-based molecular approach may aid in health monitoring and detection of MRM in a rodent colony reared for experimental purpose.

## Introduction

*Mycoplasma pulmonis* (MP) remains potentially important rodent pathogen and found in significant proportion in well-maintained animal facilities, even under strict bio-exclusion conditions. *Mycoplasma* sp. is a Gram-negative bacterium, lacking a cell wall which commonly colonize in the respiratory tract of rats [1,2] causing murine respiratory mycoplasmosis (MRM). This disease frequently goes undiagnosed due to subclinical nature. MP is transmitted vertically through the placenta and horizontally through direct contact or aerosol [3,4], and thus, it is difficult to get rid of disease without following all-in–all-out policy.

It becomes important to diagnose MP infection due to its potential interference with a wide variety of research. Commonly observed clinical signs include sneezing, nasal discharge with dyspnea or rales, and also torticollis in the case of middle/internal ear infection [4,5]. Other common signs of disease such as weight loss, hunched posture, ruffled coat, and chromodacryorrhea may also be observed [1,2]. MP commonly affects respiratory tract of animal; however, infection of the female reproductive tract is also found in some cases which may cause endometritis and perioophoritis leading to decreased fertility [5,6]. Besides the experimental interference of the organism, it also has zoonotic importance which may affect personal working with infected rats [7].

Postmortem lesions may include rhinitis, otitis, tracheitis, and bronchopneumonia. However, the severity of lesions may vary depending on a variety of host factors such as the strain, age, and immune status of animal [5,8].

Commonly used diagnostic procedures include serology, culture, histopathology, and polymerase chain reaction (PCR) [4,9]. In this study, we report a detailed study of clinical symptoms, postmortem lesions, and diagnosis of MP infection in a rat colony.

## Materials and Methods

### Ethical approval

All animal experiments were conducted in accordance with the Committee for the Purpose of Control and Supervision of Experiments on Animals (CPCSEA) guidelines.

### Housing and husbandry

Conventional colony of out-bred stocks of Wistar rat was housed in static cages under barrier conditions at government approved Animal Facility of School of Biological Sciences, NISER, Bhubaneswar, India. All the animals used during the present study were housed in polysulfone cages with corncob bedding in a controlled environment with temperature and humidity ranging between 24±3°C and 40-70%, respectively. Animals were exposed to 12 h light/12 h dark cycle as a routine practice. The present incidence of MP infection was detected during routine health monitoring of animals. Two female Wistar rats were observed to be diseased thus kept in isolation for further serological, pathological, and molecular (PCR) investigations which revealed MP-induced MRM. Thereafter, whole rat colony comprising of 36 animals was segregated based on clinical symptoms. All the animals showing symptoms of stress and disease (N=2) were sampled for PCR-based molecular diagnosis. Further 6 animals of 32 asymptomatic rats were sampled and sacrificed to study the prevalence of disease in the entire colony. All the animals were later sacrificed after the sampled animals were found to be positive for MP.

### Euthanasia

Animals were euthanized using carbon dioxide asphyxiation method as per the standard operating procedures approved by the Institutional Animal Ethics Committee of NISER, Bhubaneswar. As per the American Veterinary Medical Association and CPCSEA guidelines, CO_2_ asphyxia is an approved method of euthanasia on a wide variety of laboratory animal species including rodents [10,11]. Unlike commonly used inhalation euthanasia agents such as ethers and chloroform which are highly irritant, CO_2_ is non-irritant and widely acceptable method of euthanasia [11]. Feldman and Gupta [12] conducted a detailed study on histopathologic changes in laboratory animals resulting from various methods of euthanasia and found that euthanasia of experimental animals by overexposure to CO_2_, seemed most suitable for pulmonary studies.

### Blood collection and serum separation

About 0.5-1 ml of blood was collected directly through cardiac puncture immediately after euthanasia. Blood collected was transferred to a 1.5 ml centrifuge tube which was later centrifuged at 5000 rpm for 5 min, and serum was subsequently stored for further analysis.

### Serological diagnosis

The qualitative serological analysis was done using Sentinel Panel Test (SMART-SPOT) multiple analyte assay kit supplied by Biotech Trading Partners as per manufacturer’s instructions. The kit membrane used antigen-specific spots for 8 set of diseases as mentioned as follows:

1. KRV (Kilham rat virus)
2. MPUL (*Mycoplasma pulmonis*)
3. PVM (pneumonia virus of mice)
4. RCV/SDAV (rat coronavirus/sialodacroadenitis virus)
5. REO-3 (respiratory enteric orphan virus)
6. RPV, r-VP2 (mouse parvovirus)
7. Sendai (Sendai virus)
8. TMEV (Theiler’s murine encephalomyelitis virus).

### Gross and histopathological analysis

Detailed postmortem examination was carried out, and lesions were recorded by the veterinarian. Sections of thee lungs were fixed in ready to use buffered formalin fixative (HIMDIA S109-500ML). Fixed tissue sample was processed by routine methods in paraffin wax. Sections (5 µm) were stained with hematoxylin and eosin, and histopathologic evaluation was done by a trained veterinarian under a light microscope.

### PCR analysis

Both, tracheal washings and infected lung tissue were collected during postmortem examination and stored at −20°C. Tracheal washing was done after dissecting out trachea and lungs. The sharp end of a 26-gauge needle was cut blunt along the plastic hub and inserted into the trachea. This was secured using an artery forceps. About 0.5 ml of phosphate-buffered saline was repeatedly flushed in and out of trachea with lungs intact. DNA extraction was done using 275 µl of tracheal washing, and 20 mg of lung tissue was done using SV Genomic DNA Purification System (Promega) as per manufacturer’s instructions. DNA yield was quantified using Thermo Scientific Nano Drop 2000 spectrophotometer. 2 µl of template genomic DNA was used for running the PCR reaction. PCR was performed using species-specific primers for MP (sense primer, 5’-AGCGTTTGCTTCACTTTGAA-3’; antisense primer, 5’-GGGCATTTCCTCCCTAAGCT-3’), which generate a 266-bp amplification product. The PCR was optimised for annealing temperature. The temperature cycle followed was denaturation at 95°C for 30 s, annealing at 51°C for 30 s, and extension at 72°C for 30 s, for 40 cycles. The PCR was performed in a Bio-Rad DNA Thermal Cycler. PCR products were subjected to 1% agarose gel electrophoresis using ethidium bromide stain.

## Results

The rat colony (N=36, 6 M:29 F) was diagnosed to be symptomatic (n=2) as well as asymptomatic (n=34) carrier of *M pulmonis* infection. Animals reported positive after pathological, serological, and PCR-based molecular diagnosis were in similar age group (6-8 months) and kept under similar climatic conditions. All the animals were in the age group of 6-7 months. Based on clinical symptoms and postmortem lesions, MRM was classified as mild, moderate, and severe (Table-1). Classical symptoms of mycoplasmosis were observed in only two of the animals while the majority of the animals (about 89%) did not show any symptoms of the disease. Tiny population (N=2) showed symptoms of stress which include chromodacryorrhea and ruffled hair coat both indicative of decreased grooming and not very specific to the disease diagnosed.

**Table-1:** Classification of disease based on the severity of symptoms and lesions.

Parameters	Severity of disease		

	Mild	Moderate	Severe
Number of animals affected	34	2	2
Clinical symptoms	No observable clinical symptoms	Ruffled hair coat Porphyrin staining	Labored breathing Ruffled hair coat Porphyrin staining near eyes and nose (chromodacryorrhea) Snuffing Hunched posture Biting marks
PM lesions	Congested lungs. Some areas of red hepatization may be evident. All the lobes may or may not be involved	Multifocal to diffuse areas of red hepatization and congestion Scanty areas of gray hepatization Multiple lobes involvement	Marked diffuse areas of gray and red hepatization The whole of the lungs involved with fibrinopurulent exudates from the cut surface Multiple raised cobblestone-shaped abscess lesions over the surface

### Gross and histopathology findings

**Figure-1:**
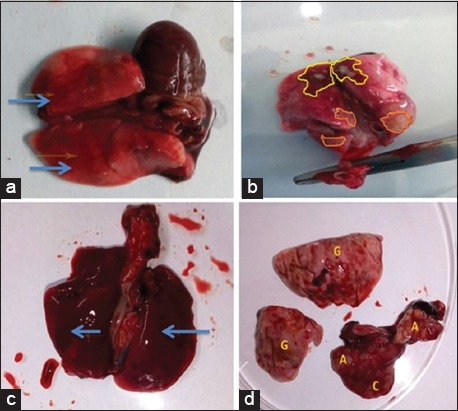
(a) Severely infected rat lung showing multiple irregular abscess (2-3 mm diameter) (A), marked areas of gray hepatization (G) which at some places are coalescing (C) with areas of red hepatization. (b) Multifocal areas of red (yellow boundary) and gray hepatization (brown boundary). Marked areas of congestion and pus-filled nodular lesions also visible grossly. (c) Lung of infected rat. Marked diffuse red hepatization of lung. (d) Diffuse congestion in lung indicated by arrow.

Gross lesions in lungs were observed on necropsy of affected animals; however, the appearance and severity of lesions varied, which were correlated with clinical symptoms, thus classified as mild (n=34), moderate (n=2), and severe (n=2). Multifocal to diffuse congestion was observed in cases classified as mild. Affected lungs had faint-to-moderate red color lesions which were firm and wet in appearance (Figure-1a). Focal areas of red hepatization were also evident in some of the lobes. Single or multiple lobes were found to be affected. No exudate was observed macroscopically over the surface as well as a cut section of the lung in this category.

Lungs of animals with a moderate severity of infection consisted of multifocal to diffuse areas of red hepatization and congestion along with focal or multifocal areas of gray hepatization having irregular margins (Figure-1b). Single to multiple abscesses were also observed on the surface of the lungs. Cut section revealed multiple abscesses with blood mixed exudate. Multiple lobes were found to be involved. These lesions indicated severe congestion along with partial necrosis in lungs of the affected animals. Necropsy examination of other case classified as moderate revealed diffuse dark red lesions indicating red hepatization whole of the lung (Figure-1c).

Entire lungs and all the lobes showed pathogenic lesion in rats which were severely affected. Marked diffuse areas of gray and red hepatization coalescing at few spots were observed. The whole of the lung was found to be involved with fibrinopurulent exudates from the cut surface. Multiple raised cobblestone-shaped firm abscesses were observed over the surface of the lungs in one of the cases (Figure-1d).

**Figure-2:**
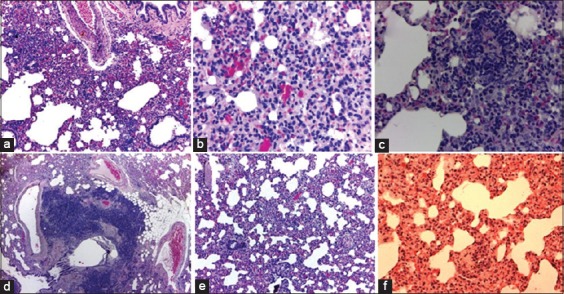
(a) Lung of rat with abscesses, inflammatory cells infiltration, and diffuse hemorrhages (10×) (indicated by arrow). (b) Severe diffuse hemorrhages lung heavily infiltrated with macrophages, lymphocytes, and neutrophils (40×). (c) Pneumonia in rat with *Mycoplasma pulmonis* disease. Marked abscess formation (40×). (d) Pneumonia in rat lung. Heavy peribronchiolar accumulation of inflammatory cells (P). Alveoli filled with inflammatory cells and mucus (4×), (e) Inflammatory infiltration and hemorrhages in alveolar septum (10×). (f) Inflammation of alveolar septum in rat lung infected with MP (40×).

Histopathological findings revealed diffuse hemorrhages along with intra-alveolar infiltrations of inflammatory cells (Figure-2a-c). Examination under high magnification showed alveoli highly infiltrated with primarily macrophages and lymphocytes along with some neutrophils (Figure-2b). Multiple small abscesses were also observed indicating necrotizing pneumonia (Figure-2d). Heavy multifocal peribronchiolar accumulation of inflammatory cells indicated chronic bronchiolitis (Figure-2d). In some sections, inflammatory cell infiltration along with hemorrhages was found along thickened alveolar septum (Figure-2d-f).

### Diagnosis

Serological test conducted in two of the animals which were initially segregated based on clinical symptoms of respiratory distress was found to be positive for MP which was indicated by colored spot.

**Figure-3:**
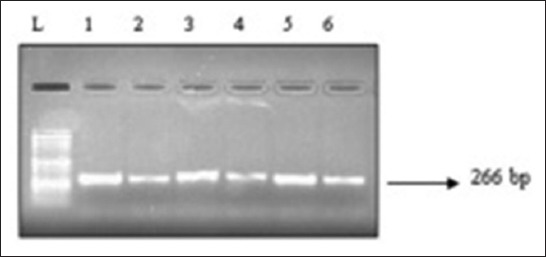
Molecular detection of *Mycoplasma pulmonis* using polymerase chain reaction: Lane L: DNA ladder of MW 1 kb. Lane 2-6: Lung tissue samples from rat lung.

Lung tissue samples and tracheal washings were PCR analyzed using species-specific primers for MP which produced bands of predicted sizes. Clearly observable band (Figure-3) confirmed the presence of MP infection in lung tissue. Genomic DNA concentration was better from lung tissue (8.2 ng/µl) than tracheal washing (2.1 ng/µl) as measured by spectrophotometer.

## Discussion

In the present study, clinical, pathological, and molecular investigations were carried out in a rat colony suspected of MP infection. MP is the cause of MRM, and its seroprevalence is high in India [13,14]. Our study indicates that severity of MP infection may vary within rat colony of the similar age group of animals housed together under the same environmental conditions. Some of the previously reported classical symptoms of MP associated respiratory tract infection such as labored breathing snuffing and hunched back [5] were observed in very few cases. Most of the animals (89%) did not show any symptoms of disease but showed pathological lesions in the lungs and were found to be PCR positive for MRM infection. No instigating factor for varying severity of disease was seen in rats. Symptoms may vary and disease may go undiagnosed in conventional animal facilities which do not adopt a stringent pathogen screening and monitoring program as the majority of the population may carry asymptomatic infection [13,14].

Distinctive morphological alterations in lungs were observed on necropsy of the affected animal. However, severity and extent of lesions varied. Severely affected animals showed severe congestion along with suppurative infection of lungs and necrotizing pneumonia. The characteristic cobblestone appearance as previously reported [3,4] was found in one of the cases indicating dilated airways containing exudate.

Moderately affected animals also showed similar lesions but with different patterns. Majority of these animals had lung lobes with diffuse red hepatization and focal areas showing gray hepatization. Hyperemia and congestion were the only findings in the lungs of most of the asymptomatic animals. Similar types of lesions have been reported with experimental infection of MP in rats [15].

MP has been also reported to cause middle ear and uterine infection [5,15]. However, in our study, none of the cases was found to have ear or uterine infection on gross examination. Our study indicates that clinical symptoms and gross pathology of lungs in MRM may vary with no distinctive correlation between varying severities of disease in different animals as we could not trace the source and timeline of disease. The previous studies reported that pathologic effects of this disease vary, depending on a variety of host, organism, and environmental factors [13].

Microscopic findings vary depending on the severity of the disease. Pulmonary congestion and hemorrhage along with abscesses formation were common in most of the cases. Massive outpourings of inflammatory cells into alveoli, along with severe hemorrhages, necrosis, and bronchiolitis were observed in severely affected lung tissue. The inflammation of interstitial septum indicating alveolitis was seen in one of the sections. Most of the histological findings were in line with gross observations and corresponded with the previous findings [2,4].

The disease was diagnosed by routinely used qualitative serological kit method [16]. The serological assay is not always considered very accurate due to the presence of cross-reacting antibodies or interfering substances that interact with assay components which may lead to false-positive results [2]. Thus, confirmatory molecular diagnosis using species-specific primers and PCR was further carried out to confirm the disease. Moreover, the use of a PCR as a diagnostic method allows the specific and rapid detection of this microorganism even in the early stage of disease [3,6].

## Conclusion

Mycoplasmosis may severely infect respiratory system of rodents and might interfere with animal research. Thus, the detailed study of clinical symptoms, pathology, and molecular approach for detection and diagnosis of this disease during health monitoring of rodent colony in a research animal facility is necessary. PCR assay may be used as a diagnostic method which allows the specific and rapid detection of this microorganism even in the early stage of the disease.

## Authors’ Contributions

The study was planned and designed by SC and SJ. BV and KM helped in sample collection and laboratory diagnosis. Interpretation of PM lesions and histopathological slides was done by SC and SJ. NS helped arranging and revising the manuscript. All authors participated in data analysis and discussion. All authors read and approved the final manuscript.
